# Hybrid neutrosophic enhanced MobileNetV2 model for leukemia blood cell classification

**DOI:** 10.3389/frai.2026.1763872

**Published:** 2026-04-10

**Authors:** V. B. Prahaladhan, Megha Suhanth, L. Jani Anbarasi, T. Ashika, V. Vishruth Reddy, R. Anvesh Reddy

**Affiliations:** 1 School of Computer Science and Engineering, Vellore Institute of Technology, Chennai, India; 2 Department of Mathematics, School of Advanced Sciences, Vellore Institute of Technology, Chennai, India

**Keywords:** contrast limited adaptive histogram equalization, deep learning models, MobileNetV2, neutrosophic framework, VGG16, wavelet shrinkage denoising

## Abstract

**Introduction:**

Leukemia is a type of cancer that originates in the bone marrow, causing uncontrolled production of abnormal white blood cells that disrupt normal blood function and weaken the immune system. Manual inspection is time-consuming and error-prone, relying heavily on the expertise and experience of medical professionals.

**Methods:**

The proposed study presents a hybrid model for classifying leukemia by integrating transfer learning and neutrosophic domain enhancement. Neutrosophic domain transformation splits the RGB channel image into Truth (T), Falsity (F), and Indeterminacy (I) components to address uncertainty, ambiguity, and poor contrast in blood cell representations. This enables the improvement of features more directly linked to leukemia identification. The images are augmented using wavelet sharpening and contrast-limited adaptive histogram equalization (CLAHE) on the T component, total variation minimization (TVM) on the F component, and wavelet shrinkage denoising on the I component.

**Results:**

This framework was trained and tested on the Leukemia Blood Cell Image Classification dataset, which included 3,256 peripheral blood smear (PBS) images across 4 classes: Benign, Early, Pre, and Pro. A transfer learning architecture based on MobileNetV2 was used for classification, and training was conducted using a 70:15:15 split for training, validation, and testing, respectively. The proposed neutrosophic-enhanced MobileNetV2 model achieved an overall testing accuracy of 98.36% and a macro F1-score of 0.98, demonstrating significant enhancement in multi-class leukemia classification.

**Discussions:**

The incorporation of the neutrosophic enhancement method significantly improves classifier performance, resulting in higher accuracy without increasing computational power.

## 1 Introduction

Leukemia, a blood malignancy characterized by the abnormal proliferation of white blood cells (Terwilliger and Abdul-Hay, 2017), remains a major global health concern with more than 400,000 new cases reported annually. Early detection is important, as timely diagnosis significantly improves survival outcomes since treatment begins in the initial stages. Leukemia diagnosis depends on the microscopic examination of blood smears, which is time-consuming and requires the expertise of trained pathologists (Bain, 2005). Artificial intelligence (AI) has gained considerable importance across various medical fields, such as liver tumor segmentation (Litjens et al., 2017), breast cancer detection (Esteva et al., 2017), brain tumor segmentation (Zhang et al., 2001), and hematological malignancies (El Alaoui et al., 2022). Advancements in AI have also created possibilities for automating leukemia detection (Shafique and Tehsin, 2018) and classification, achieving better performance compared to traditional machine learning-based feature extraction techniques (Mohapatra et al., 2014; Fadzil et al., 2010). The integration of deep learning (DL) approaches for leukemia diagnosis effectively addresses the limitations of conventional methods through automated, accurate, and efficient detection.

Several architectures, such as MobileNetV2 (Sandler et al., 2018), ResNet (Khalaf et al., 2016), transfer learning models (Sahoo et al., 2022), transformer networks (Islam et al., 2024), and attention mechanisms (Tiwari et al., 2018), have shown better performance in a wide range of medical image classifications. Accuracy in leukemia detection is affected by poor-quality microscopic blood smear images, which include noise, blurred cell boundaries, and low contrast. This becomes critical when distinguishing between different stages of leukemia, which play a vital role in accurate assessment with subtle variations in cell morphology. To address these limitations, a hybrid framework is proposed that incorporates neutrosophic set theory (NST; Smarandache, 2003) to enhance images through contrast enhancement, noise reduction, and cell boundary refinement, thereby preserving elite morphological features. The contributions of the proposed model include the following:

- It provides a mathematical framework that permits the representation of uncertainty in image pixels in terms of three elements (T, I, and F), which can precisely capture the inherent indeterminacy of cell boundaries in blood smear images.
- Converting images into the neutrosophic domain can enhance feature extraction, enabling the extraction of previously indistinguishable features, and most importantly, the minor variations that differentiate various stages of leukemia.
- Directly measures noise and ambiguities in the image to enable specifically targeted enhancement that removes noise without removing critical diagnostic features.
- The complementary nature of the T and F components allows edge sharpening, with cellular boundaries becoming more defined, resulting in enhanced white blood cell segmentation.

The remaining sections of the paper are as follows: The literature review is presented in Section 2, and the proposed methodology and architectures used for leukemia classification are discussed in Section 3. Section 4 includes performance metrics used to assess the performance of the model. Section 5 details the experimental setup implementation results. Section 6 concludes future research opportunities regarding the applicability of NST to other datasets or fields.

## 2 Literature review

Pavithra (2023) proposed a dual-efficient MobileNetV2 architecture for detecting acute lymphoblastic leukemia (ALL) from blood smear images using a dataset of 3256 images collected from 89 patients categorized into early Pre-B, Pre-B, and Pro-B ALL. The model attained 98.88% training accuracy, 98.58% testing accuracy, a precision of 0.98, and an F1-score of 0.98. Sulaiman et al. (2023) introduced a hybrid method, ResRandSVM, for ALL identification using blood smear images. The features are extracted using seven pre-trained deep learning models called ResNet152, VGG16, DenseNet121, MobileNetV2, InceptionV3, EfficientNetB0, and ResNet50. Significant features are then selected using ANOVA and principal component analysis (PCA) and are classified using Random Forest (RF) and Support Vector Machine (SVM), where SVM attains better accuracy. The integrated method enhanced both the efficiency and accuracy of ALL detection.

Khalifa et al. (2021) explored the impacts of neutrosophic set theory (NST) on improving deep transfer learning models for COVID-19 classification using chest X-ray images. The authors proposed a framework that transforms grayscale X-ray images into the neutrosophic domain, generating three independent sets: indeterminacy set (I), truth membership set (T), and falsity membership set (F) to address challenges associated with limited datasets. These neutrosophic components were then used as input to pre-trained models, including AlexNet, GoogLeNet, and ResNet18, to classify images into four categories: normal, COVID-19, bacterial pneumonia, and viral pneumonia. Among all components, the indeterminacy set (I) achieved the best performance, attaining a classification accuracy of 87.1% over 36 training epochs with varying train and test splits.

Salama et al. (2024) analyzed NST enhances medical images by reducing noise, thus improving contrast by mapping them into the neutrosophic fuzzy domain. Different enhancement operations are applied to each component independently, and the components are classified using an SVM classifier and segmented using the K-means algorithm. This approach achieved an accuracy of 98% by accounting for the falsity and indeterminacy values. Kaur and Lal (2020) analyzed a comprehensive review of neutrosophic logic in biomedical image processing, highlighting the superiority of NL over traditional methods for managing uncertainties in medical images, particularly in tasks such as denoising, segmentation, and classification. By effectively addressing ambiguity and indeterminacy (I) in medical data, NST has shown the potential to significantly improve diagnostic accuracy.

Abdullah (2024) improved breast cancer classification by integrating deep learning models with neutrosophic set theory. Breast cancer images were converted into the neutrosophic domain and separated into the T, I, and F components. Seven pre-trained deep learning models were evaluated using accuracy, precision, recall, and F1-score. The results demonstrated that models trained in the neutrosophic domain outperformed others trained in the fuzzy domain, confirming that the incorporation of deep learning with NST enhances classification precision and reliability (Guo et al., 2009).

Sert and Avci (2019) introduced the NS expert maximum fuzzy-sure entropy method for the automatic detection of glioblastoma tumor boundaries in MRI images. By integrating fuzzy sure entropy with NST, the method provided more accurate boundary detection than NS with Otsu thresholding, SVM, Fuzzy C-Means (FCM), and Darwinian particle swarm optimization (DPSO). Wady et al. (2020) developed an intelligent framework that combines NST with slantlet transform (SLT) to enhance texture-based feature extraction in MRI images, achieving an accuracy of 98.94% and an AUC of 0.99. Cheng et al. (2011) performed a segmentation technique that integrates NST with an improved fuzzy C-means (IFCM) algorithm. By reducing indeterminacy within the neutrosophic domain, this study achieved highly accurate segmentation of challenging, noisy medical images. Ghanbari et al.^1^ reviewed the application of neutrosophic logic in image processing, highlighting its strengths in managing uncertainty, thus improving segmentation accuracy by preserving essential image features and enhancing image retrieval. Himel et al. (2024) proposed a feature fusion-based ensemble framework integrating EfficientNetB7 and MobileNetV3Large for acute leukemia diagnosis, achieving a multi-class accuracy of 99.3% across ALLIDB1, ALLIDB2, and ASH datasets. This ensemble model demonstrates the effectiveness of deep feature fusion for improving classification accuracy and performs conventional preprocessing. Ghanbari Talouki et al. (2025) analyzed existing image and video data, including diffusion, patch-based, and deep-learning approaches, to preserve temporal consistency and handle uncertainty in moving objects, using neutrosophic set theory for robust segmentation. Dhatchinamoorthy et al. (2025) applied a neutrosophic-based semantic segmentation algorithm to the CASIA V1 iris image dataset with Gaussian and Poisson noise, achieving an accuracy of 85%. Fan et al. (2025) proposed a neutrosophic set-based defect detection for CSP LED images that models uncertainty, suppresses noise through similarity and enhancement operations, and accurately detects defective LED beads with an accuracy of 99%. The summary of the related study is given in Table 1. Existing studies have demonstrated better performance using deep learning and transfer learning architectures for leukemia classification, but still has some limitations in classification when the images have subtle modifications in the pattern (Jawahar et al., 2024). Many approaches directly processed the raw microscopic images without explicitly addressing image uncertainty, staining variations, low contrast, and noise present in peripheral blood smear images. The existing models depend on large annotated datasets and may struggle to generalize when subtle morphological variations exist between leukemia stages. Furthermore, most conventional techniques apply uniform preprocessing operations without considering pixel-level ambiguity or indeterminacy features in medical images. Although neutrosophic set theory (NST) has been explored in other medical imaging domains, its application to multi-class leukemia classification remains limited, particularly in combination with lightweight transfer learning architectures. These constraints motivated the development of the proposed hybrid neutrosophic enhancement framework that explicitly models uncertainty and performs component-wise enhancement to improve discriminative feature representation prior to classification.

**Table 1:** Summary of key studies integrating NST in medical image analysis.

References	Application area	Methods/models used	NST usage	Dataset/images	Performance/key outcomes
Pavithra (2023)	Leukemia ALL classification	Dual efficient MobileNetV2	Normal images	3,256 images	Accuracy: 98.88%
Sulaiman et al. (2023)	ALL detection	ResRandSVM	Normal images	Blood smear images	Improved accuracy
Khalifa et al. (2021)	COVID-19 X-ray classification	AlexNet GoogLeNet ResNet18	NST-based process	Chest X-ray images	Accuracy: 87.1%
Salama et al. (2024)	Medical image classification	SVM and k-means	Neutrosophic fuzzy domain	Medical images	Accuracy: 98%
Abdullah (2024)	Breast cancer classification	Seven pretrained DL models	Neutrosophic domain	Breast cancer images	Improved accuracy
Sert and Avci (2019)	Glioblastoma edge detection	NS-EMFSE	NST combined with entropy	100 MRI images	Jaccard Index: 0.965
Wady et al. (2020)	Brain tumor diagnosis	NST + Slantlet transform	NST	MRI images	Accuracy: 98.94%
Cheng et al. (2011)	Medical image segmentation	Improved fuzzy C-means	NST	Complex and noisy medical images	Improved accuracy

## 3 Materials and methods

This paper analyzed a neutrosophic-enhanced image preprocessing technique for leukemia cell classification. The microscopic blood smear images are converted into Truth (T), Indeterminacy (I), and Falsity (F) components. These neutrosophic components are then preprocessed using contrast-limited adaptive histogram equalization (CLAHE), wavelet-based sharpening, unsharp masking, and total variation minimization to enhance contrast, suppress noise, refine edges, and preserve morphological structures across each RGB channel. The enhanced T, I, and F components are subsequently reconstructed to form high-quality images with improved feature clarity and discriminative features. The reconstructed images are analyzed using the MobileNetV2 model pretrained on ImageNet to efficiently classify four leukemia classes: Benign, Early, Pre, and Pro.

### 3.1 Neutrosophic set theory-based image enhancement

**Figure 1:**
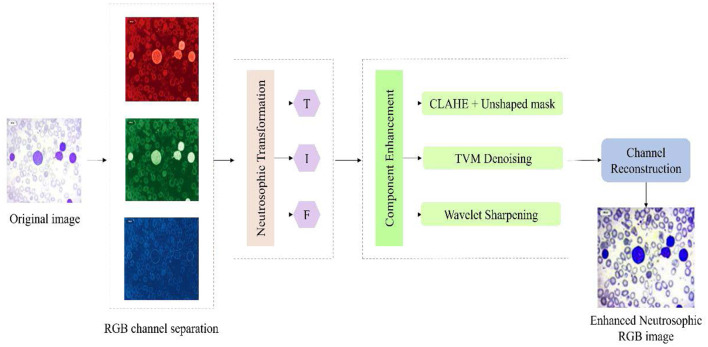
Neutrosophic image enhancement process.

NST is a mathematical framework for modeling uncertainty, vagueness, and indeterminacy. Each pixel is converted into three components: truth (T), falsity (F), and indeterminacy (I). The T component indicates the degree to which a pixel belongs to a relevant region of interest, while the F component shows the extent to which it does not belong. The I component captures the ambiguity related to the pixel arising from noise overlapping cell boundaries, staining variations, or low-contrast regions. This process improves global and local contrast texture and strengthens morphological features that are critical for segmentation, edge detection, and classification. By reducing ambiguity in the I component and reinforcing meaningful information in the T and F components, NST-based enhancement significantly improves the quality and reliability of leukemia cell analysis. The overall workflow of the NST-enhanced image enhancement process is shown in Figure 1.

Each input image is transformed into the neutrosophic domain, yielding three components per pixel. The first component, T, represents the degree to which the pixel intensity reflects actual brightness and is calculated by normalizing the original pixel value as shown in Equation 1.


$$\begin{array}{l} T\left(x,y\right)=\frac{g\left(x,y\right)}{g_{max}} \end{array}$$ (1)

where *g*(*x, y*) represents the original pixel intensity and *g*_max_ is the maximum intensity value that is 255. The second component *F* measures the degree of non-membership or falsity and is computed as the complement of the Truth value 1−*T* as shown in Equation 2.


$$\begin{array}{l} F\left(x,y\right)=1-T\left(x,y\right) \end{array}$$ (2)

The third component *I* captures the uncertainty or noise associated with the pixel and is determined by calculating the local standard deviation within a moving window of a specified size, as described in Equation 3.


$$\begin{array}{l} I\left(x,y\right)=\frac{\sigma \left(x,y\right)}{\sigma_{max}} \end{array}$$ (3)

where σ(*x, y*) is the local standard deviation computed over a window *w* centered at pixel (*x, y*) as described in Equations 4, 5, σ_*max*_ represents the images' highest standard deviation value.


$$\begin{array}{l} \sigma \left(x,y\right)=\sqrt{\frac{1}{\left|w\right|}*\sum_{\left(x,y\right)}^{W}{\left(g\left(x,y\right)-\mu \left(x,y\right)\right)}^{2}} \end{array}$$ (4)

$$\begin{array}{l} \mu \left(x,y\right)=\frac{1}{\left|W\right|}*\sum_{\left(x,y\right)}^{w}g\left(x,y\right) \end{array}$$ (5)

### 3.2 Component enhancement

Neutrosophic representation of Truth (T), Falsity (F), and Indeterminacy (I) of the microscopic ALL images is enhanced using specialized preprocessing techniques. These enhancement operations are designed to improve contrast, suppress noise, and strengthen the morphological and edge-related features required for ALL analysis. By individually optimizing the T, I, and F components, the proposed framework results in a more discriminative and diagnostically reliable image representation for ALL classification.

Truth Enhancement:

Contrast-limited adaptive histogram equalization was applied to enhance the contrast of the T component using an 8 × 8 tile grid with a clip limit of 2.0. This was followed by unsharp masking with a radius and amount of 1.5, optimized specifically for cytological image analysis, to sharpen the image details. The enhancement is mathematically represented in Equation 6.


$$\begin{array}{l} T_{enhanced\;}=CLAHE\left(T\right)+\;\alpha *\left(T-G_{\sigma }\left(T\right)\right) \end{array}$$ (6)

where *G*_σ_ represents a Gaussian blur operation, standard deviation as σ, and α is the sharpening factor. This operation enhanced local contrast while preserving the important structural features in the T component.

Falsity Enhancement:

Total variation minimization (TVM) denoising is applied to reduce noise while preserving edges. This is achieved by optimizing a function that balances noise reduction while maintaining the original image features using a regularization parameter (λ) set as 0.1.


$$\begin{array}{l} F_{enhanced}=\arg min_{u}\left\{\lambda \;*TV\left(u\right)+\frac{1}{2}\;||u-F|{|}^{2}\right\} \end{array}$$ (7)

Equation 7 enhanced the F component by finding the optimal *u* that minimizes a combination of two terms. The first term *TV*(*u*) controls the smoothness of the enhanced component across the image, while the second term ||u – F||^2^ ensures closeness to the original F component. The parameter λ balances smoothing with fidelity, resulting in a denoised *F* component with well-defined edges.

Indeterminacy Enhancement:

Indeterminacy I component is enhanced by utilizing a two-stage wavelet-based approach. Wavelet shrinkage denoising is first applied to eliminate uncertainty without losing significant structural features. The BayesShrink algorithm, which operating in soft mode, is adaptively suppresses noise based on the statistical properties of the wavelet coefficients, ensuring that only uncertain or noisy components are attenuated without degrading meaningful features. This algorithm adaptively computes thresholds based on the statistical characteristics of wavelet subbands to efficiently remove noise, as shown in Equation 8.


$$\begin{array}{l} I_{denoised}=\;W^{-1}\left(T_{BayesShrink}\left(W\left(I\right)\right)\right) \end{array}$$ (8)

where *W* and *W*^−1^ are the forward and inverse wavelet transforms, and *T*_*BayesShrink*_ is the BayesShrink thresholding operator. After denoising, the multiscale wavelet sharpening is applied to enhance edge clarity and fine structural features. This process decomposes the images into multiple frequency sub-bands using Daubechies-2 (db2) wavelets with two levels of decomposition. The detail coefficients at each scale are then amplified using a sharpening factor of 1.3, while the β parameter is set to 1 to control the non-linear boosting of high-frequency information. This sharpening enhances subtle textures and boundary transitions by selectively increasing the magnitude of detail coefficients, and its mathematical formulation is represented in Equation 9.


$$\begin{array}{l} I_{enhanced}=\;W^{-1}\left(\left\{c_{0},\;\beta_{1}c_{1},\;\ldots ,\;\beta_{n}c_{n}\right\}\right) \end{array}$$ (9)

Coefficient *C*_0_ represents the approximation lowest frequency coefficient and is set to 0 where *C*_1_ to *C*_*n*_ represent the detail coefficients are represented at different scales. During sharpening, the detail coefficients are selectively amplified by raising them to a scale-dependent sharpening factor β_*i*_ that is greater than unity. In the proposed study, the value of the sharpening factor is set to 1.3, as it provides controlled enhancement of high-frequency features strong enough to improve edges and fine textures.

### 3.3 Image reconstruction

After enhancing all three components, the enhanced neutrosophic subsets are then fused to reconstruct the final enhanced image using Equation 10.


$$\begin{array}{l} g^{\prime }\left(x,y\right)=T_{enhanced}\left(x,y\right)-F_{enhanced}\left(x,y\right)+\;I_{enhanced}\left(x,y\right) \end{array}$$ (10)

The reconstruction ensures an effective balance among components, where the augmented T component reinforces features confidently present in the image, the F component suppresses regions confidently identified as non-features or background, and the I component adds refined features from ambiguous, transitional, or boundary regions. Together, these fused results in an image with improved clarity, structural consistency, and enhanced feature visibility suitable for ALL classification. The augmented truth component highlighted the significant features, such as the cell nuclei, cytoplasmic patterns, and other morphological features essential for classification. The F component effectively removed background noise, and the I component preserved regions such as cell borders, membrane boundaries, and internal structural variations. Empirical fine-tuning of weighted parameters is performed to further optimize the reconstruction quality as represented in Equation 11.


$$\begin{array}{l} g^{\prime }\left(x,y\right)={\alpha \;*T}_{enhanced}\left(x,y\right)-{\beta \;*F}_{enhanced}\left(x,y\right)+\\ {\gamma \;*I}_{enhanced}\left(x,y\right) \end{array}$$ (11)

**Figure 2:**
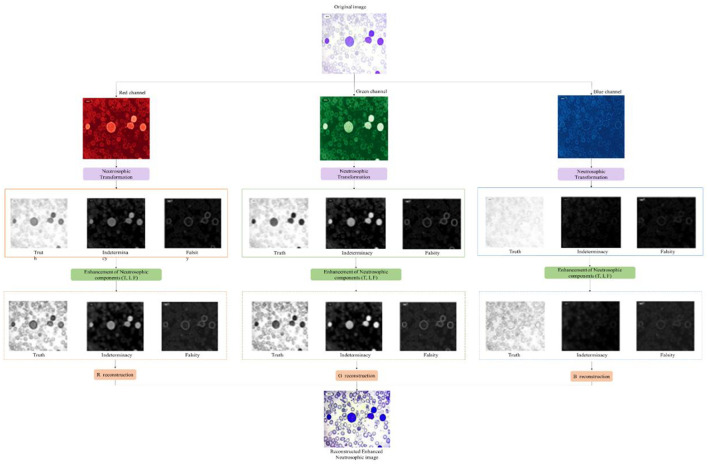
Overall neutrosophic enhancement workflow applied to a sample image.

The optimization parameters α, β, and γ are set to 1. These weights allowed the reconstruction process to capture relevant features by suppressing unrelated background regions. RGB images were generated from the R, G, and B channels, confirming natural color information and enhancing structural features. These images provided better preservation of critical diagnostic features, reduced noise in uniform regions, and improved edge sharpness around cell boundaries. The sample enhancement and reconstruction outputs are shown in Figure 2.

### 3.4 MobileNetV2 architecture

The neutrosophic-enhanced generated images were resized to 224 × 224 pixels and normalized to the range [0,1] to reduce illumination inconsistencies and stabilize network training, as shown in Equation 12.


$$\begin{array}{l} I_{norm}\left(x,y\right)=\;\frac{I\left(x,y\right)}{255} \end{array}$$ (12)

This study adopts a transfer learning strategy using MobileNetV2, selected for its lightweight design, fast inference, and strong representational performance, all of which are crucial for medical image analysis. The architecture includes an input tensor to process enhanced leukemia images of size 224 × 224, as shown in Equation 13.


$$\begin{array}{l} I_{input}=\;R^{224\times 224\times 3} \end{array}$$ (13)

The model was first processed using robust feature extraction with pre-trained weights from ImageNet, with the top classification layers removed to customize for the new task. All base-model layers are frozen at this stage to retain the premium learned features, allowing the model to recognize general patterns without modification during early training. The pre-trained MobileNetV2 (without its top layers) is used to extract high-level semantic features as shown in Equation 14. Each convolutional layer of the base model is frozen during initial training as given in Equation 15.


$$\begin{array}{l} F={MobileNetV2}_{base}\left(I_{input}\right) \end{array}$$ (14)

$$\begin{array}{l} \theta_{base}=constant \end{array}$$ (15)

A GlobalAveragePooling2D layer marks the end of the feature extraction pipeline, replacing the traditional flattening operation to minimize the parameter count while sustaining spatial relationships within the features. The GlobalAveragePooling2D layer reduces the number of parameters and preserves spatial relationships as shown in Equation 16, resulting in a compact feature *z* from the feature maps.


$$\begin{array}{l} z_{i}=\frac{1}{H\times w}\sum_{x=1}^{H}\sum_{y=1}^{W}F_{i}\left(x,y\right) \end{array}$$ (16)

A dense layer with 128 neurons and ReLU activation provides non-linear feature transformation as given in Equation 17.


$$\begin{array}{l} h=ReLU\left({W1}_{z}+\;b_{1}\right) \end{array}$$ (17)

The final dense layer contains four neurons, each representing the Benign, Early, Pre, and Pro ALL classes. The probability distribution over all classes is computed using SoftMax, as shown in Equation 18, where the predicted label ĉ is computed as given in Equation 19.


$$\begin{array}{l} P\left(c_{i}|h\right)=\;\frac{e^{W_{2,i}h+b_{2,i}}}{\overset{4}{\underset{j=1}{\sum }}e^{W_{2,j}h+b_{2,j}}} \end{array}$$ (18)

$$\begin{array}{l} ĉ=\arg\;max_{i}\;P\left(c_{i}h\right) \end{array}$$ (19)

**Figure 3:**
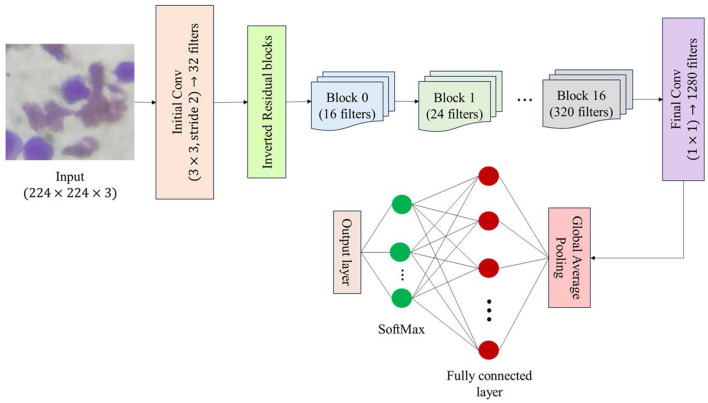
Architectural overview of the MobileNetV2.

This proposed hybrid architecture transforms high-dimensional neutrosophic-enhanced image data into elite feature representations to obtain probabilistic class predictions. The flow of the neutrosophic data-enhanced MobileNetV2 pipeline is illustrated in Figure 3.

### 3.5 Adam optimizer

Adam (Adaptive Moment Estimation) was used as the optimization algorithm due to its ability to adaptively adjust the learning rate for each *trainable parameter* θ in the network, including convolutional filters, biases, batch normalization parameters, and fine-tuned MobileNetV2 layers. For the gradient of the loss with respect to a parameter θ at iteration *t* denoted as *g*_*t*_, Adam computes the exponential moving averages of the first moment and second moment using Equation 20.


$$\begin{array}{l} m_{t}=\beta_{1}m_{t-1}+\left(1-\beta_{1}\right)g_{t},\;mv_{t}=\beta_{2}v_{t-1}+\left(1-\beta_{2}\right){g_{t}}^{2} \end{array}$$ (20)

with β_1_ and β_2_ represent the decay rates for the respective moment estimates. Bias-corrected estimates are then obtained as given in Equation 21.


$$\begin{array}{l} \hat{m_{t}}=\;\frac{m_{t}}{1-\beta_{1}^{t}},\hat{v_{t}}=\;\frac{v_{t}}{1-\beta_{2}^{t}} \end{array}$$ (21)

The parameters are updated as given in Equation 22, where each trainable parameter θ is adjusted as given below.


$$\begin{array}{l} \theta_{t+1}=\;\theta_{t}-\alpha \frac{\hat{m_{t}}}{\sqrt{\hat{v_{t}}}+\;\epsilon } \end{array}$$ (22)

where α denotes the learning rate and ϵ is a small constant ensuring numerical stability. This adaptive mechanism enables Adam to effectively manage noisy, sparse, or complex gradients, which commonly arise in medical image classification and fine-tuning of deep transfer learning models.

### 3.6 Loss function

Categorical cross-entropy (Sampathila et al., 2022) was used as the loss function due to its suitability for multiclass classification tasks. The leukemia samples were categorized into four distinct classes, and the loss function measures the divergence between the true class distribution and the predicted probability distribution produced by the SoftMax layer. Let *y*_*i*_ denote the true probability of class *i* (represented in one-hot encoded form) and ŷ represent the predicted probability for class *i*. The categorical cross-entropy loss is defined in Equation 23.


$$\begin{array}{l} L_{CE}=-\sum_{i=1}^{c}y_{i}\log\left({ŷ}_{i}\right) \end{array}$$ (23)

where *c* denotes the total number of classes. The gradient properties of the loss ensure stable and effective training across all classes for consistent classification of leukemia types. The overall algorithm is detailed in Algorithm 1.

Algorithm 1 Proposed neutrosophic-based deep transfer learning framework. 
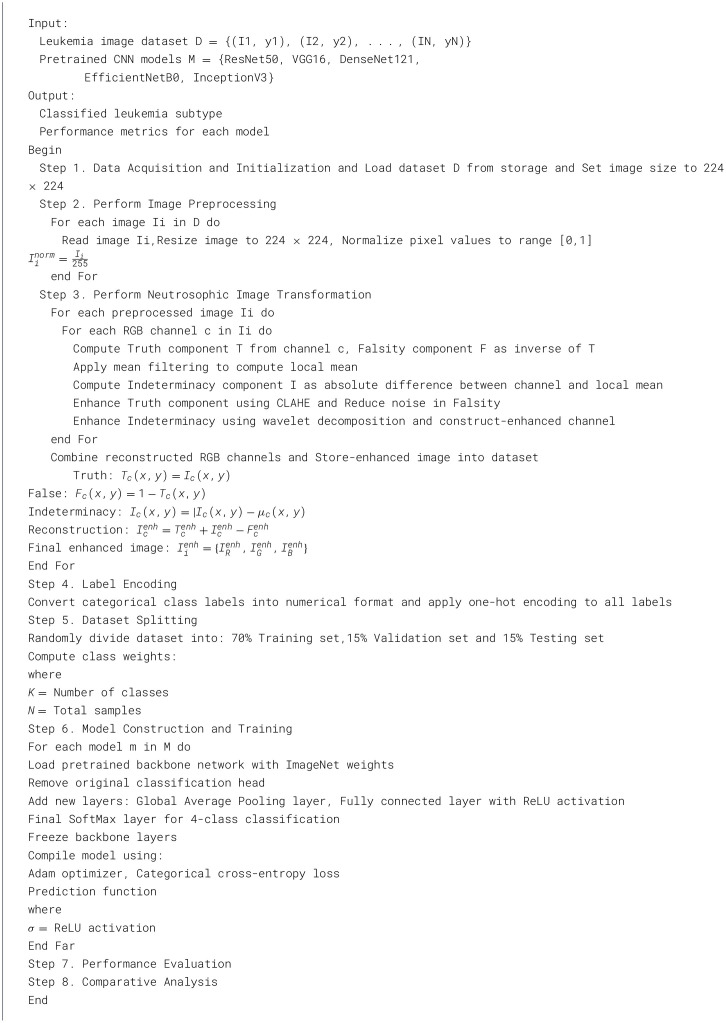

## 4 Performance metrics

The neutrosophic-enhanced leukemia classification is evaluated using performance metrics to measure its effectiveness across four classes: Benign, Early, Pre, and Pro. These metrics enabled a thorough assessment of the model's performance and highlighted the extent to which the neutrosophic enhancement technique improves overall diagnostic accuracy. Accuracy shows the proportion of correctly predicted samples and helps track learning behavior during the training and validation phases, while test accuracy provides an unbiased measure of generalization, as defined in Equation 24.


$$\begin{array}{l} Accuracy=\;\frac{TP+\;TN}{TP+TN+\;FP+\;FN\;} \end{array}$$ (24)

where *TP, TN, FP, and FN* represents true positives, true negatives, false positives, and false negatives, respectively. *TP* refers to images correctly classified as belonging to a specific leukemia class. *TN* denotes images correctly identified as not belonging to a given class. *FP* represents images incorrectly classified as belonging to a class when they actually belong to other leukemia types or benign cases. *FN* corresponds to images that truly belong to one of the leukemia categories (Benign, Early, Pre, or Pro) but are misclassified by the neutrosophic-enhanced model.

Precision measures the correctness of positive predictions for each class by measuring the quantity of predicted positives that are truly positive, as defined in Equation 25. Higher precision indicates fewer false positives, ensuring that other leukemia types are rarely misclassified as the target class.


$$\begin{array}{l} Precision=\;\frac{TP}{TP+\;FP\;} \end{array}$$ (25)

Recall measures the models' ability to identify all true instances of a specific class, as shown in Equation 26. Higher recall reflects effective detection of true positives, which is essential in medical diagnosis, where false negatives carry significant clinical risk.


$$\begin{array}{l} Recall=\;\frac{TP}{TP+\;FN\;} \end{array}$$ (26)

The F1-score, as shown in Equation 27, is the harmonic mean of precision and recall, providing a balanced metric that accounts for both false positives and false negatives.


$$\begin{array}{l} F1\;Score=2^{*}\frac{Precision*Recall}{Precision+Recall} \end{array}$$ (27)

## 5 Results and discussion

The experimental setup, dataset, and augmentation techniques are detailed along with the neutrosophic transformation, which maps microscopic images into the neutrosophic domain as the basis for subsequent processing. The neutrosophic enhancement modules and their outputs are detailed along with how the processed neutrosophic components are recombined to reconstruct enhanced images.

### 5.1 Experimental setup

The experimental environment used Google Colab Pro with an NVIDIA GTX 1,050 GPU, 16 GB RAM, and Python 3.7. TensorFlow 2.8 was used for model development, OpenCV 4.5 for image processing, PyWavelets for multi-resolution analysis, and scikit-image for advanced image manipulation. The neutrosophic image enhancement process required an average of 0.8 s per image. The MobileNetV2 model was trained for 50 epochs using the Adam optimizer with a learning rate of 0.001 and a batch size of 32.

### 5.2 Dataset preparation and augmentation

**Figure 4:**
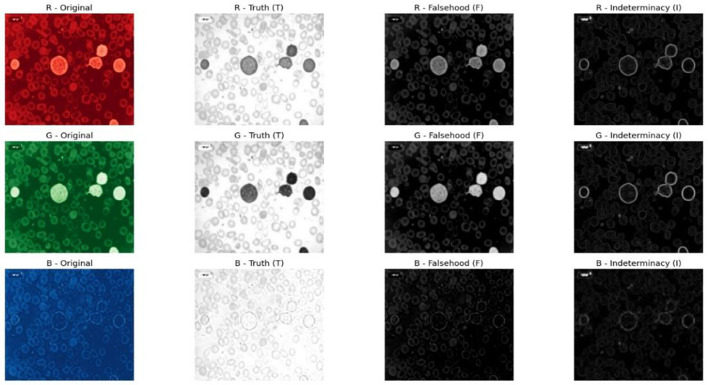
Sample neutrosophic transformation of an image.

The dataset consisted of 3,256 high-quality PBS images collected from 89 patients categorized into four leukemia classes (Ghaderzadeh et al., 2022): (Aria et al., 2021) Benign, Early, Pre, and Pro. To address class imbalance and increase variability, augmentation techniques such as 90 °, 180 °, and 270 ° rotations, as well as horizontal and vertical flips, were applied using Ubuntu-based Python libraries. This produced a balanced dataset with approximately 985 images per class, totaling 3,940 images. Figure 4 illustrates a sample neutrosophic transformation demonstrating how the original microscopic image is decomposed into its neutrosophic components for further enhancement.

Data augmentation was applied to correct class imbalance and improve model robustness. Few classes were expanded using horizontal flips (*Ih*′), vertical flips (*Iv*′) and rotations of 90 °, 180 °, and 270 °. This process increased the number of smaller classes to match the largest class, resulting in a balanced distribution of about 985 images per class. The original images were preserved to maintain data authenticity. Each transformation *T* applied to an image *I* can be represented mathematically using Equations 28–32:


$$\begin{array}{l} Ih^{\prime }=I\left(W-x-1,y\right) \end{array}$$ (28)

$$\begin{array}{l} Iv^{\prime }\;=I\left(x,H-y-1\right) \end{array}$$ (29)

$$\begin{array}{l} Ir^{90^{\prime }}\;=I\left(y,W-x-1\right) \end{array}$$ (30)

$$\begin{array}{l} Ir^{180^{\prime }}\;=I\left(W-x-1,\;H-y-1\right) \end{array}$$ (31)

$$\begin{array}{l} Ir^{270^{\prime }}\;=I\left(H-y-1,x\right) \end{array}$$ (32)

where:

- *I*(*x, y*) = intensity of the pixel at coordinates (x,y) in the original image.
- *I*′(*x, y*) = intensity of the pixel at coordinates (x,y) in the transformed image.
- *W* = width of the image (number of horizontal pixels).
- *H* = height of the image (number of vertical pixels).

### 5.3 Neutrosophic transformation and enhancement

**Figure 5:**
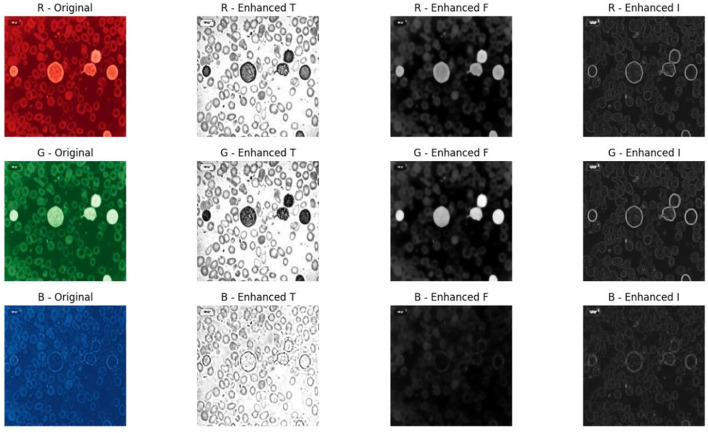
Neutrosophic images after enhancement.

Neutrosophic transformation of leukemia cell images clearly broke the image down into three components. Experimental data revealed that component F emphasized the background area, while boundary and texture details were only highlighted by component I, and component T reported only primary cell structures. Neutrosophic image enhancement I part was denoised and sharpened using the wavelet transform, while component F was denoised using total variation minimization. Contrast and edge improvement in the T component were achieved through CLAHE combined with unsharp masking. As shown in Figure 5, enhancement techniques made good improvements in contrast, edge definition, and noise reduction compared to the basic neutrosophic transformation.

### 5.4 Image reconstruction

**Figure 6:**
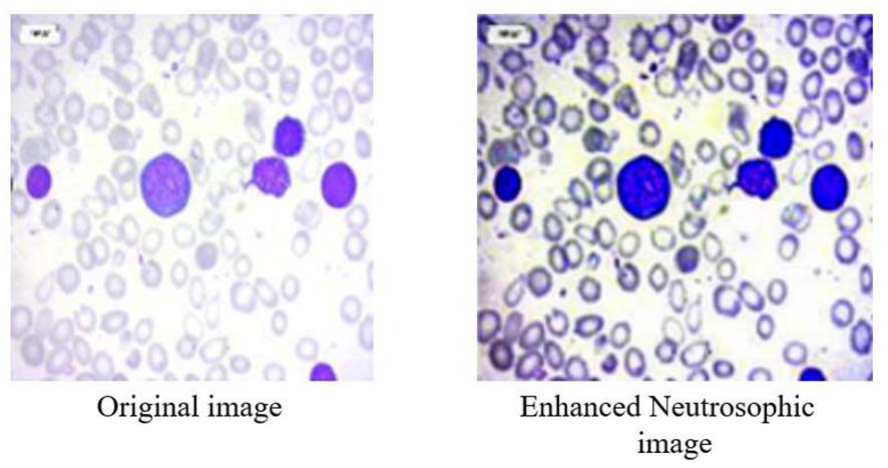
Sample reconstructed images.

Reconstructed images exhibited significant improvements in visual quality, with cell boundaries became sharper and clearly defined. These enhancements preserved the original structural and morphological features of the leukemia cells, ensuring that no diagnostically relevant information was altered or lost during processing. The neutrosophic reconstruction not only improved visual clarity but also maintained the integrity of the original cell information, enabling accurate classification and analysis. Images reconstructed showed sharper cell boundaries, enhanced contrast, and reduced background noise while preserving the original morphological structures of leukemia cells. Figure 6 highlights the improvements obtained through the neutrosophic enhancement and reconstruction process.

**Figure 7:**
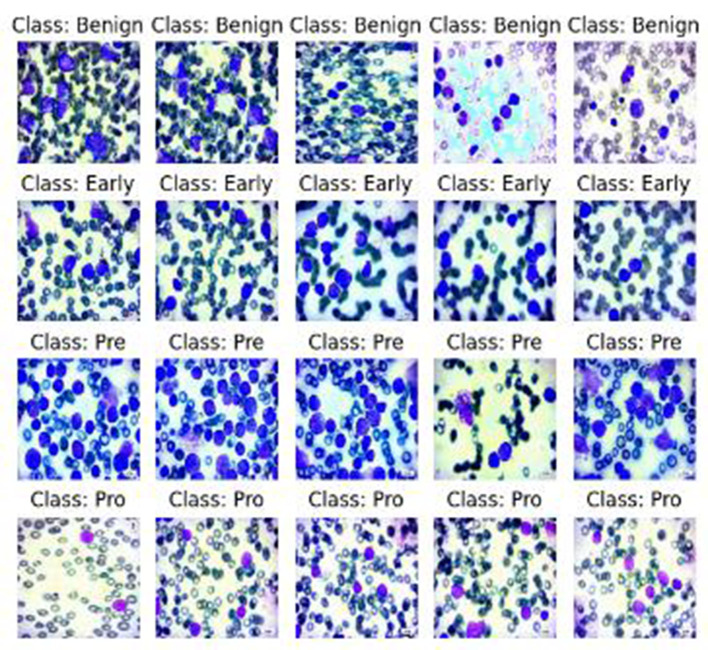
Enhanced images.

**Figure 8:**
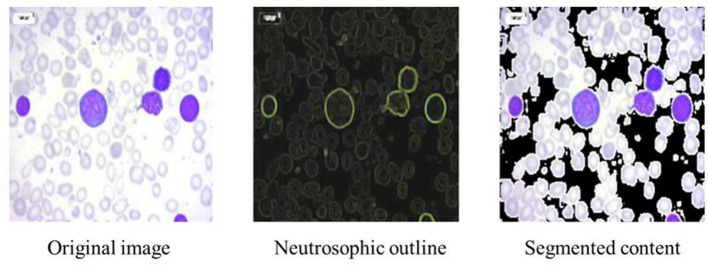
Reconstructed and segmented I component.

Figures 7, 8 show the neutrosophic-enhanced reconstructed sample images. Figure 7 details the overall enhancements in contrast, edge definition, and noise reduction, while Figure 8 presents the reconstructed and segmented I component, which highlights cellular boundaries, chromatin distribution, cytoplasm delineation, and nuclear structures that are important for accurate leukemia stage classification.

### 5.5 Model performance evaluation

The leukemia class labels were processed into a one-hot encoded vector. The dataset was split into training, validation, and testing as 70%, 15%, and 15% to ensure sufficient data for learning while enabling evaluation on independent samples. The performance metrics were computed exclusively on the testing set. Training was performed up to 50 epochs with a batch size of 32 to optimize memory usage and maintain stable gradient updates. The model weights corresponding to the highest validation accuracy were restored automatically. During training, the loss and accuracy metrics were continuously monitored during both the training and validation processes through their learning curves, facilitating assessment of convergence and potential overfitting, as shown in Table 2. The MobileNetV2 model was compiled using Adam optimizer with a learning rate of 0.001. Adam was selected for its adaptive learning rate capabilities, combining the benefits of AdaGrad and RMSProp, which effectively handle noisy or sparse gradients common in medical image classification. The moderate learning rate preserved the pretrained ImageNet features during fine-tuning, ensuring stable convergence. Learning curves indicated steady improvement in training and validation accuracy, with validation accuracy rising from 96.62% to 98.98%, and training plateauing after approximately 20–35 epochs. The model achieved a loss of 0.03 and an accuracy of 0.98, demonstrating stronger generalization capability in the test dataset. Detailed evaluation, including precision, recall, and F1-scores, confirmed the effectiveness of the model, validating both the architectural selection and the neutrosophic enhancement.

**Table 2:** Training and validation performance of the analyzed model.

Model	Accuracy	Loss
ResNet50	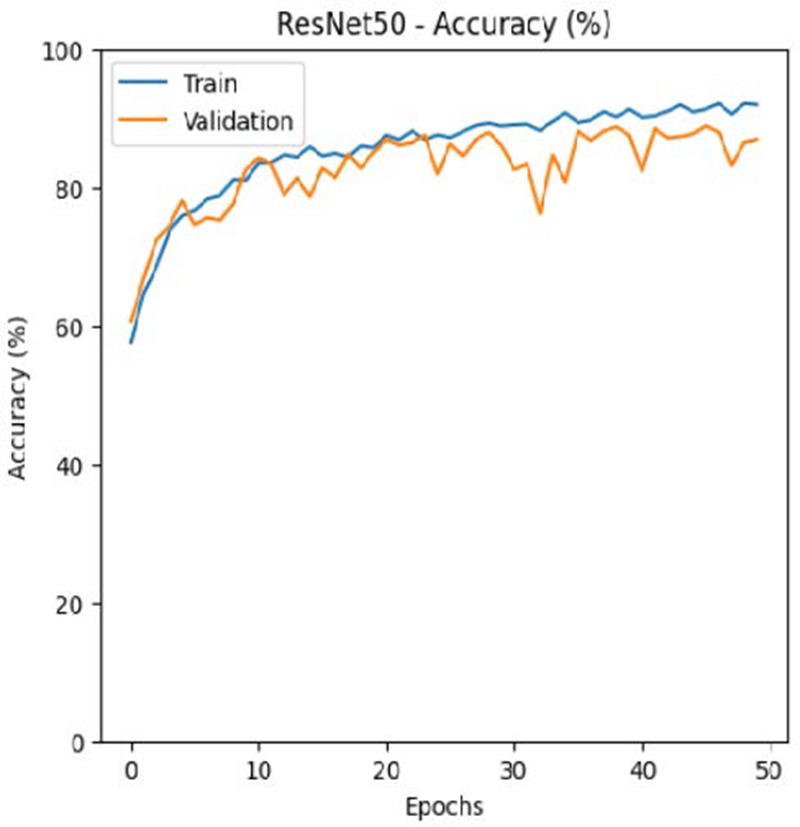	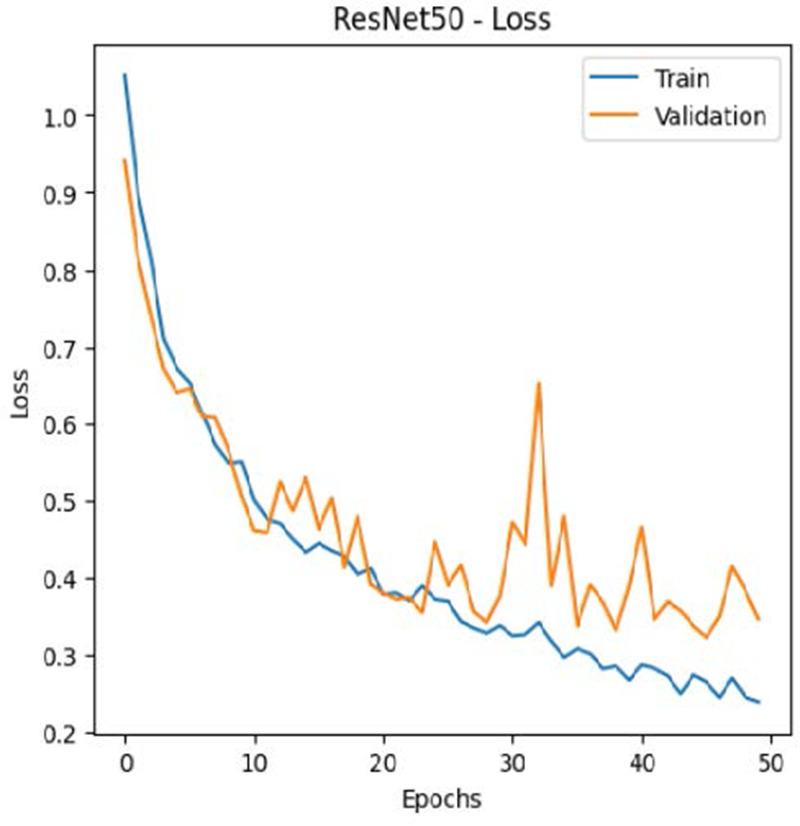
VGG16	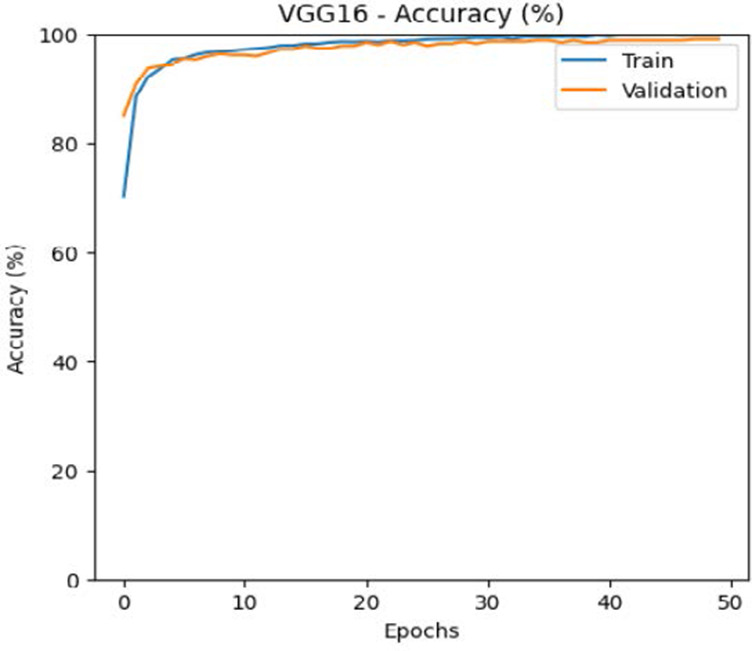	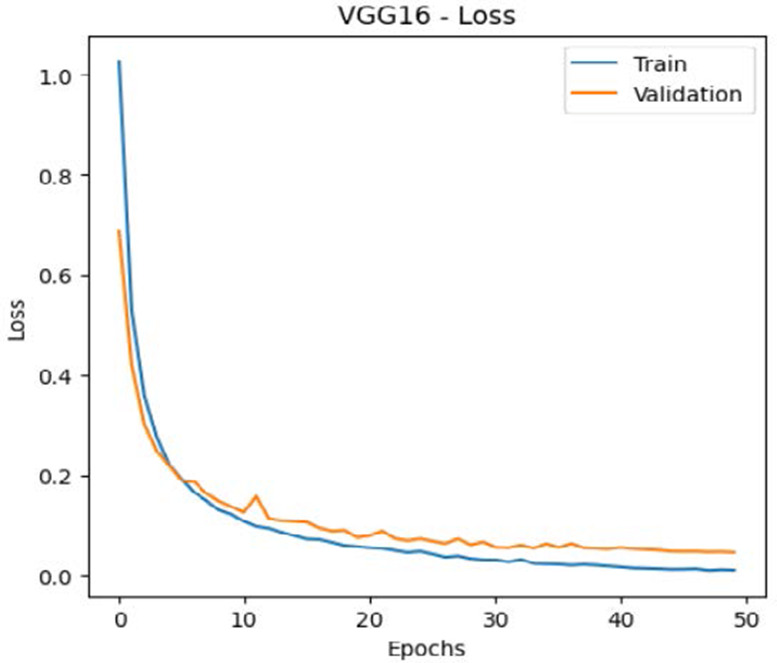
EfficientNetB0	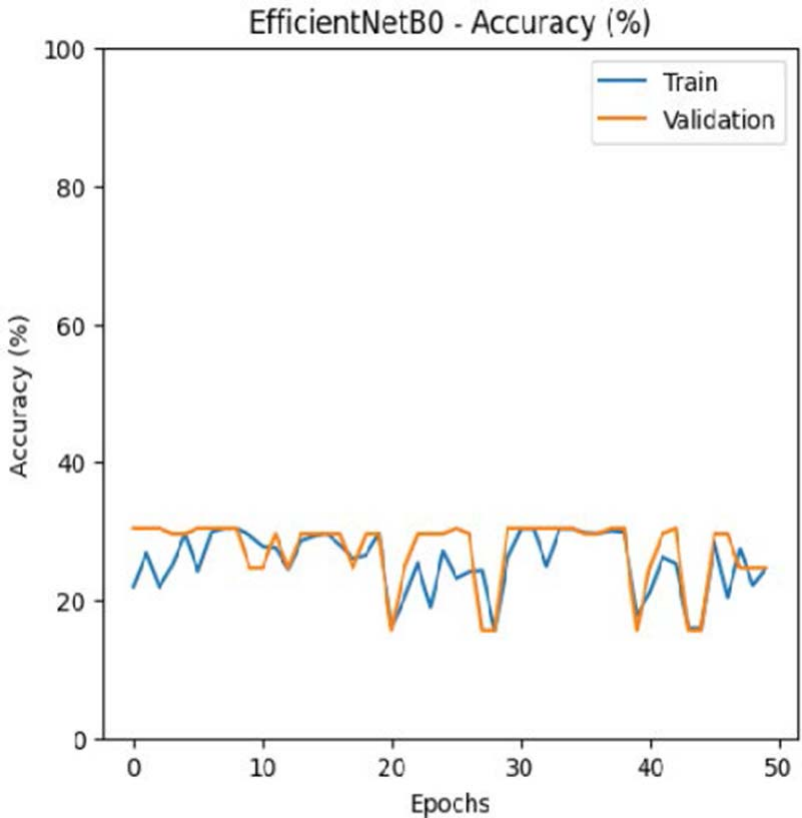	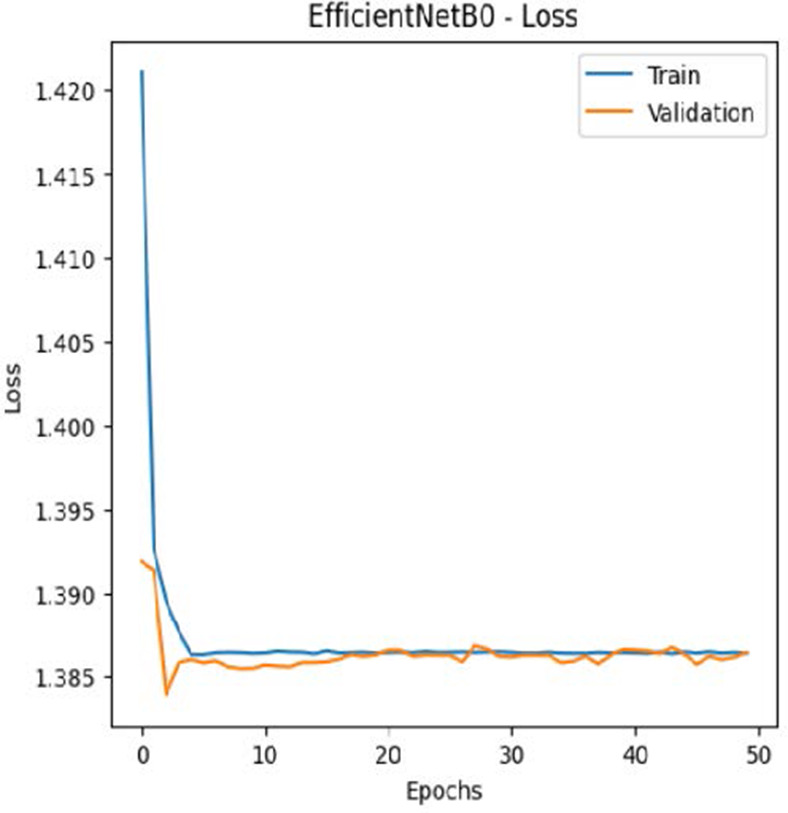
InceptionV3	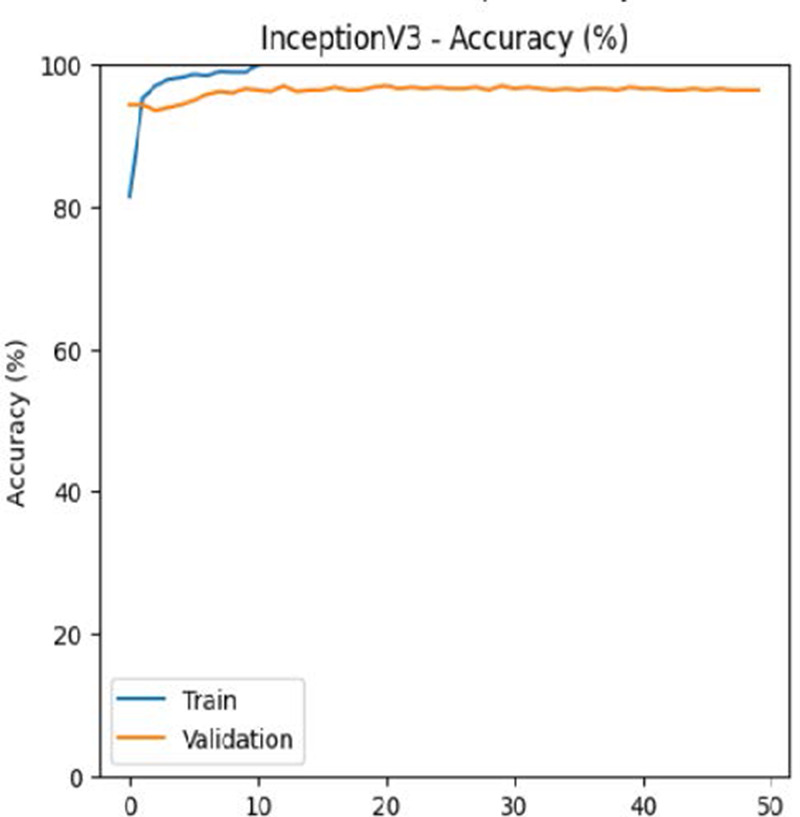	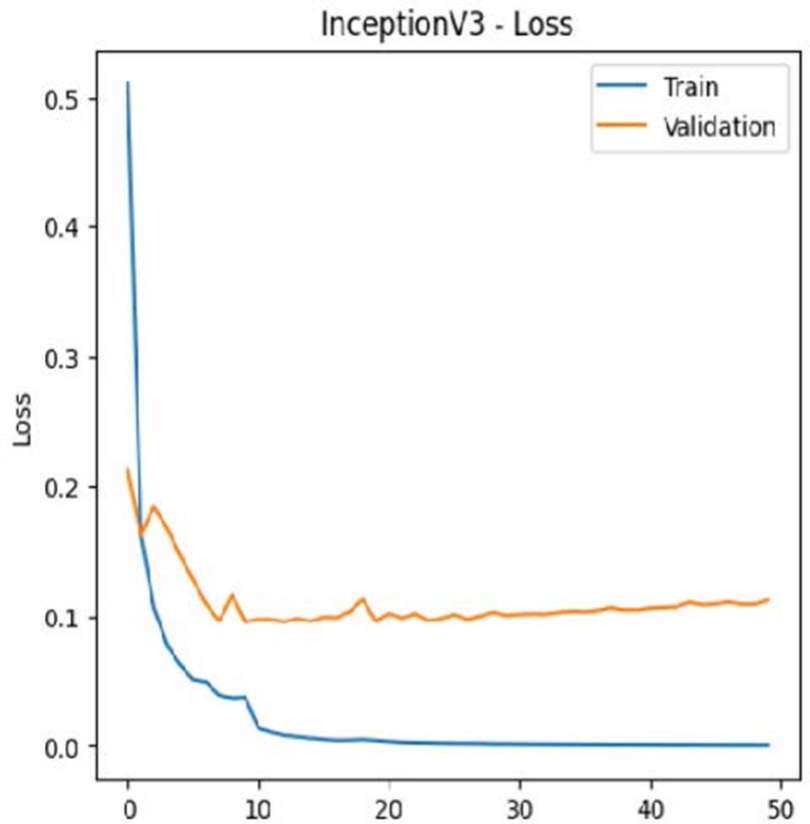
MobileNetV2	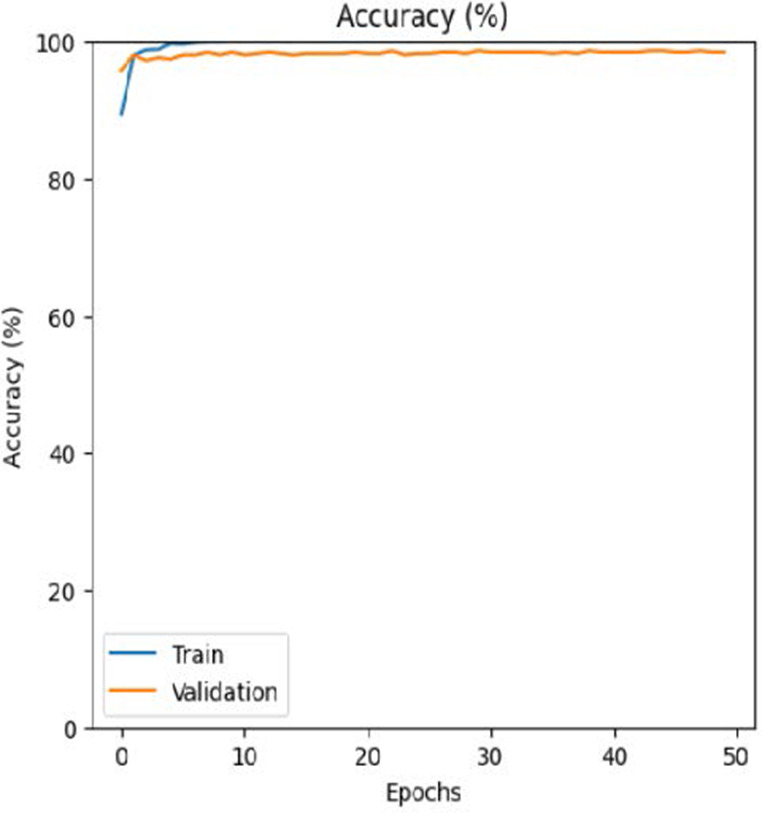	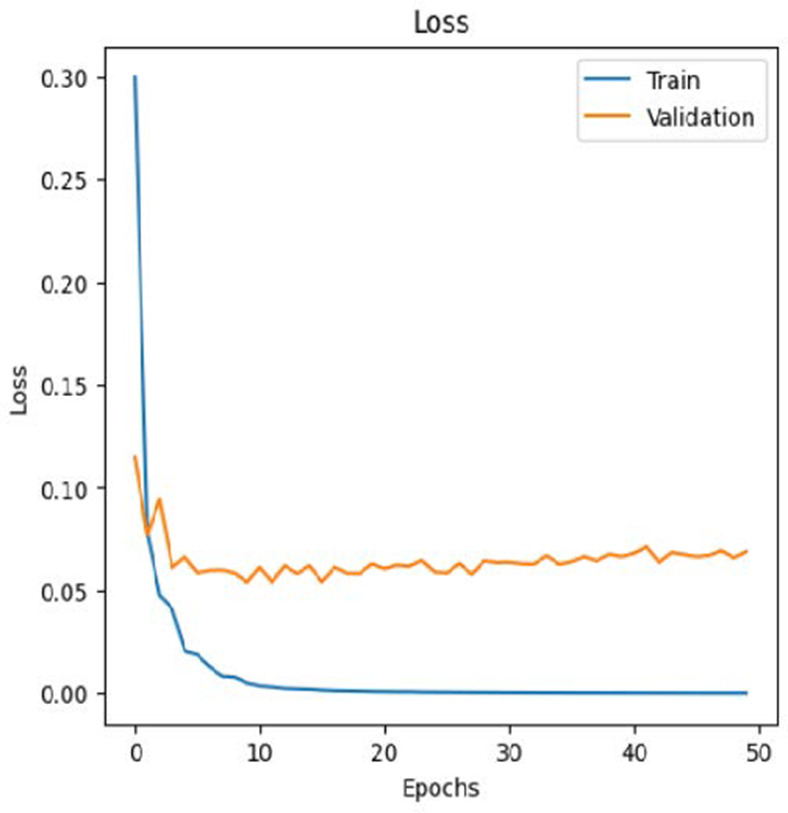

The proposed neutrosophic-enhanced MobileNetV2 model achieved an overall accuracy of 98.36%. The Pro class achieved a precision of 1.00, while the Pre, Benign, and Early classes attained F1-scores of 0.99 and 0.98, showing high sensitivity and specificity, as shown in Table 3. The class distribution and consistent performance highlight the robustness and practical applicability of the model to healthcare areas.

**Table 3:** Per-class performance metrics.

Class	Precision (%)	Recall (%)	F1-score (%)
Benign	0.96	0.97	0.97
Early	0.97	0.99	0.98
Pre	1.00	0.97	0.99
Pro	0.99	1.00	1.00
Macro avg	0.98	0.98	0.98
Weighted avg	0.98	0.98	0.98
Overall AUC	0.99		
Accuracy	98.36		

The MobileNetV2 model achieved a test accuracy of 98.36%. The Pro class achieved a higher F1-score and precision, while the other classes maintained F1-scores above 0.98. The confusion matrix and the ROC plot are shown in Table 4.

**Table 4:** Confusion matrix and the ROC plot for the multi-class classification.

Model	Confusion matrix	ROC plot
ResNet50	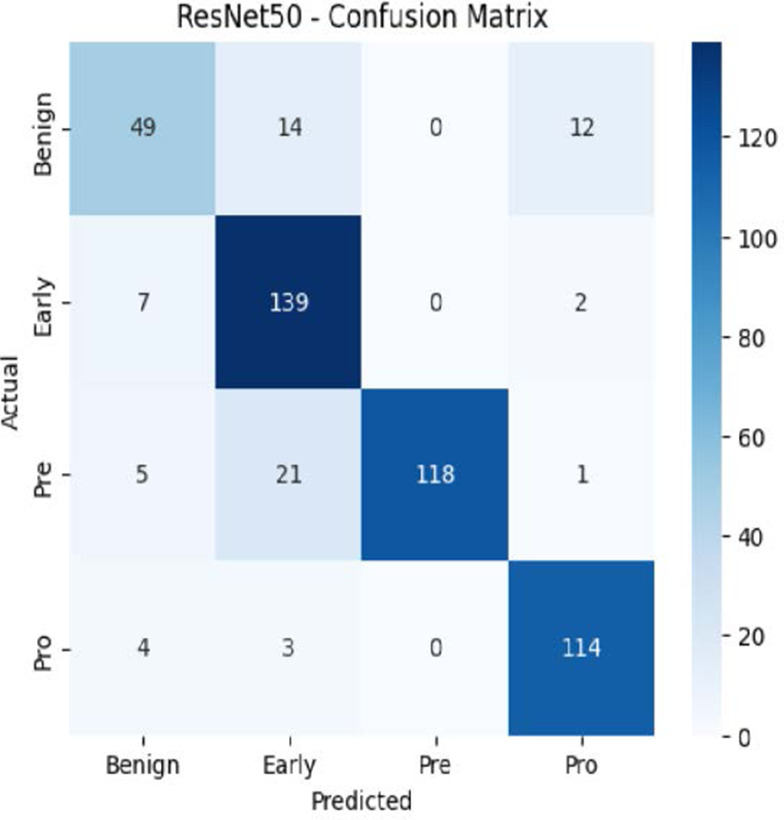	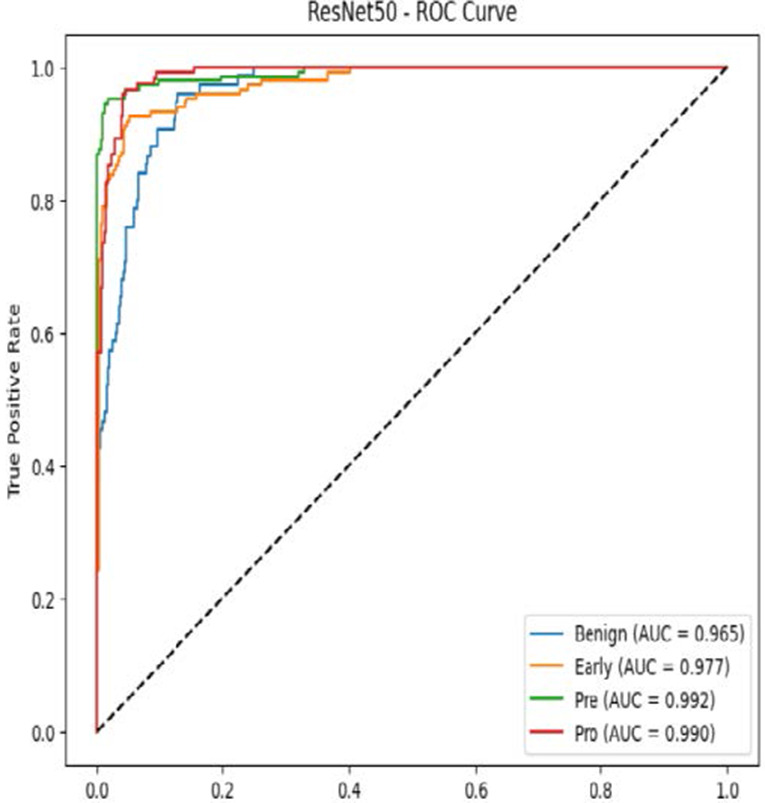
VGG16	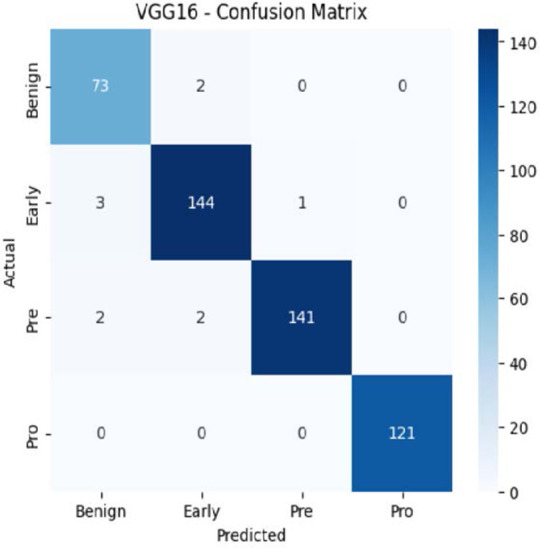	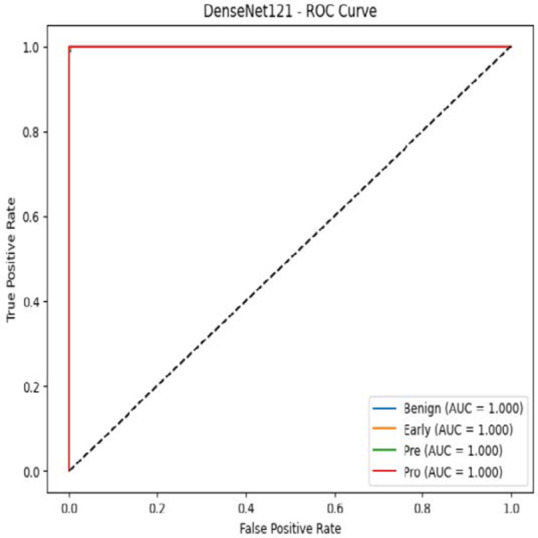
EfficientNetB0	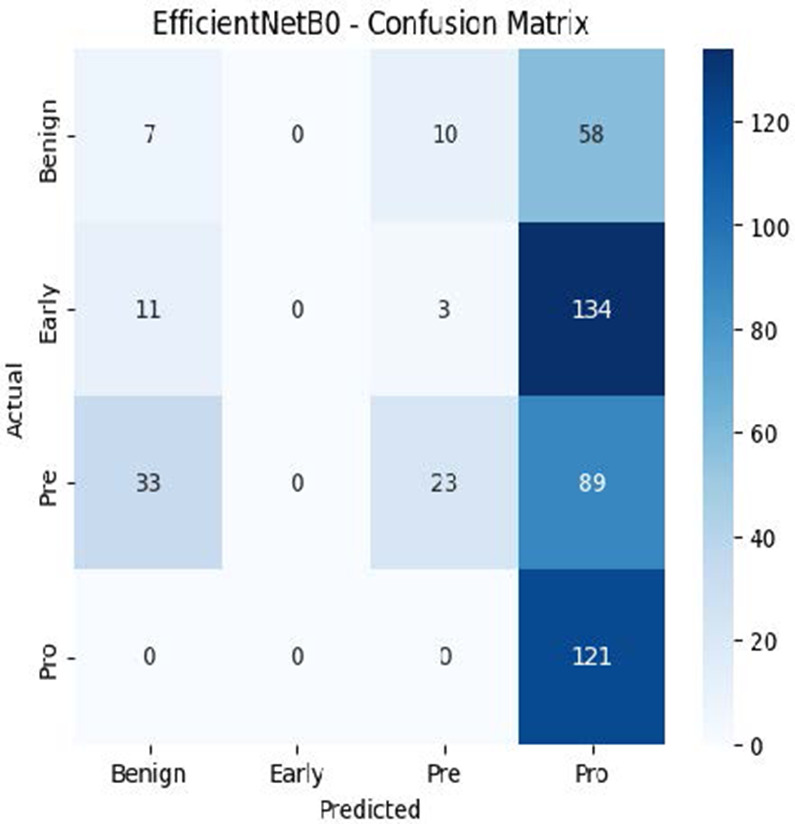	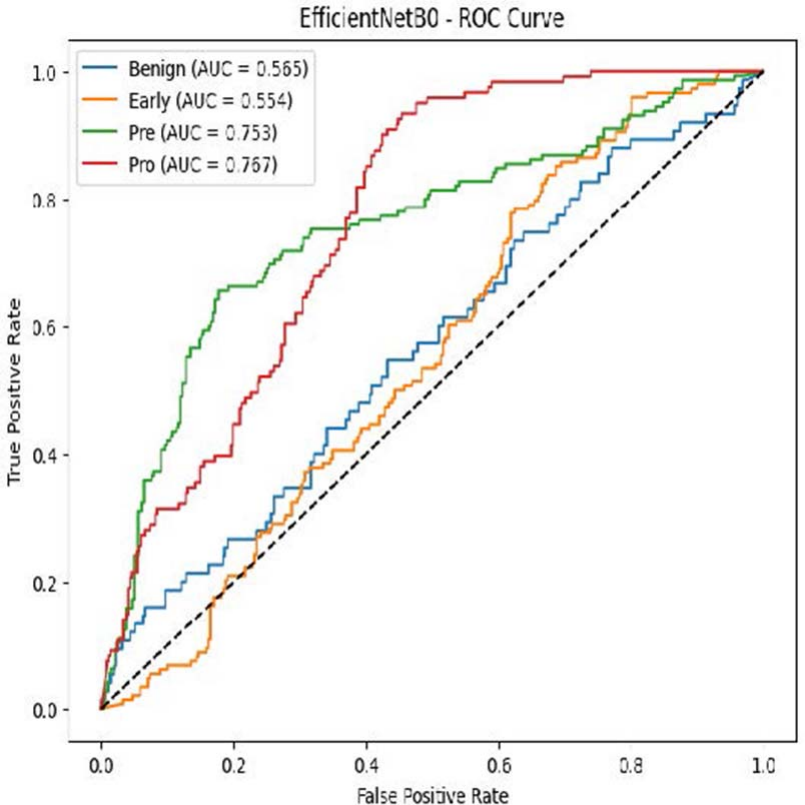
InceptionV3	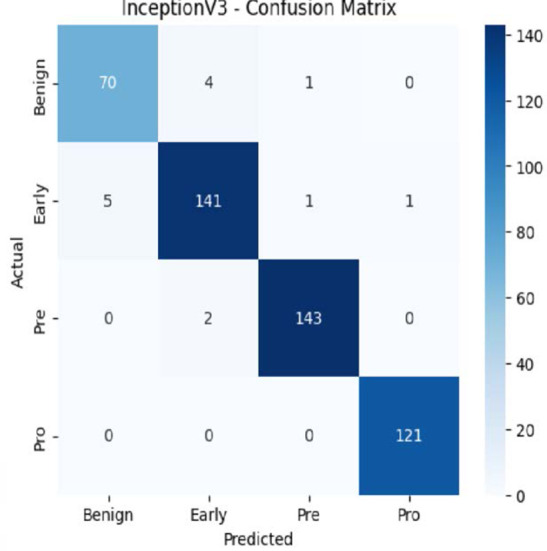	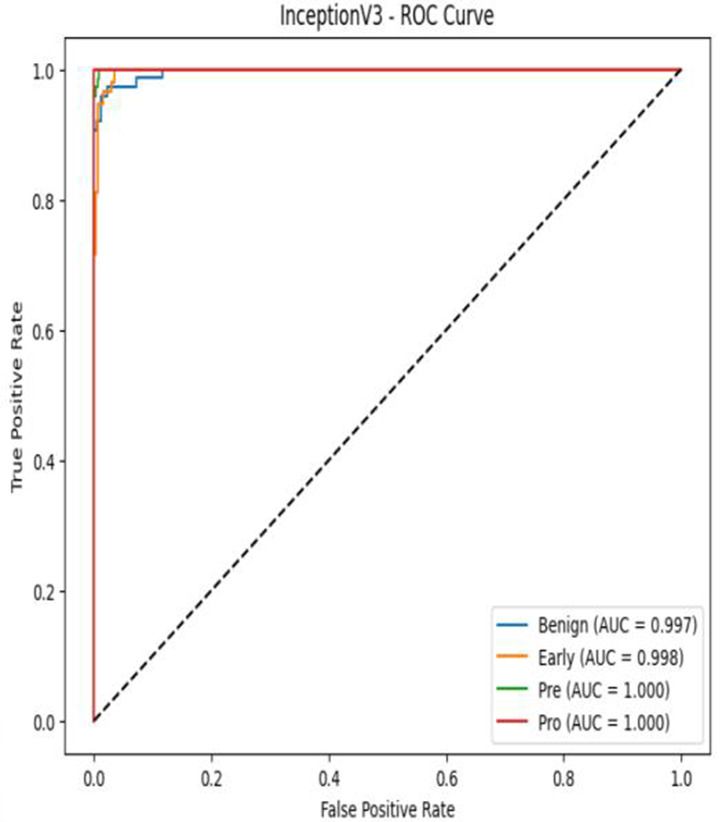
MobileNetV2	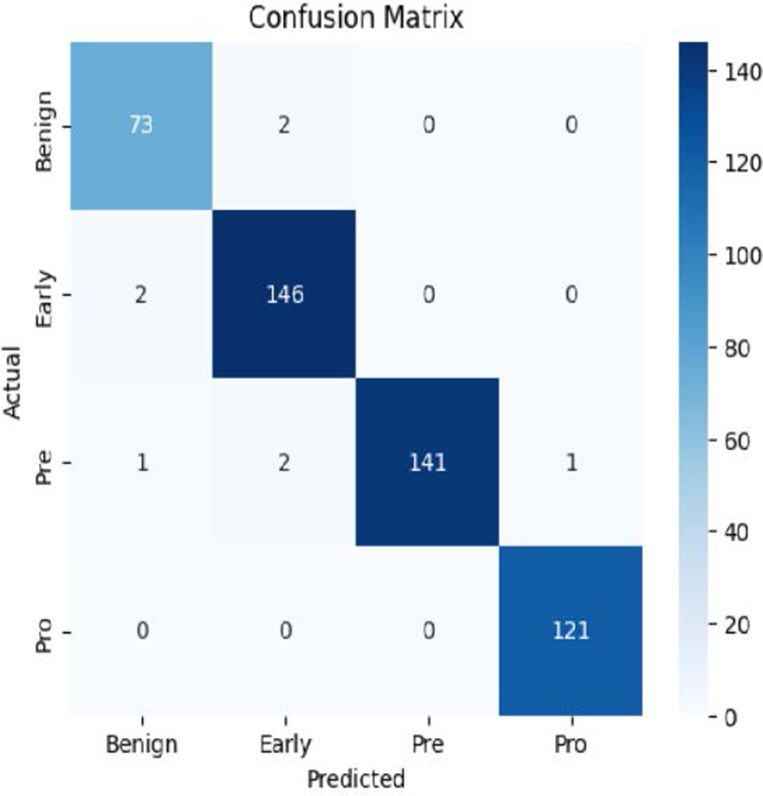	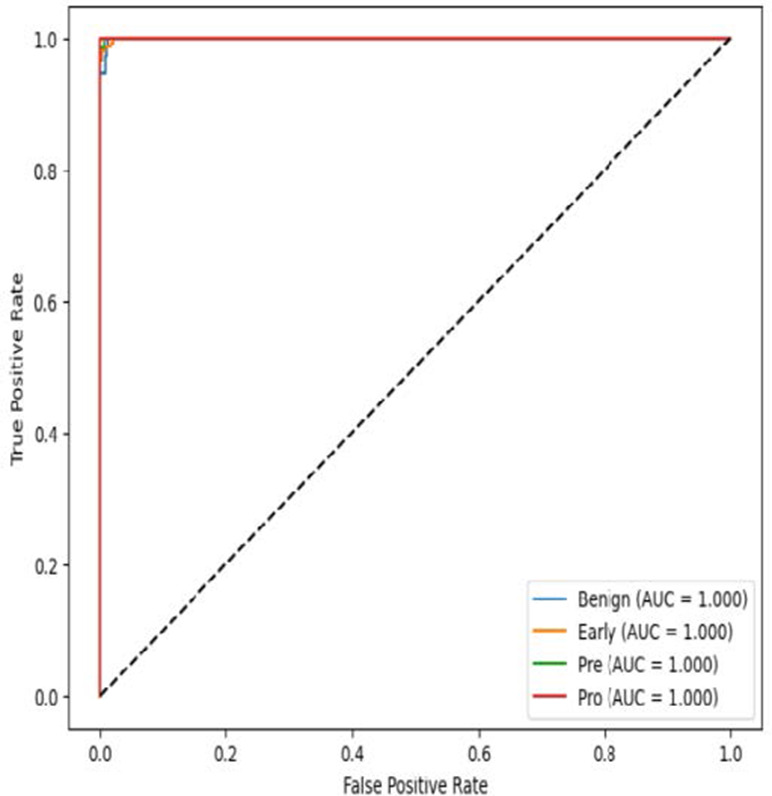

**Figure 9:**
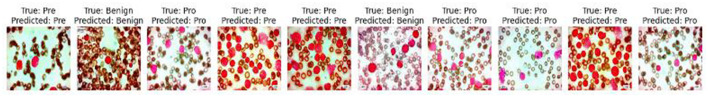
Prediction results.

The preservation of high accuracy without overfitting shows that neutrosophic enhancement effectively extracted discriminative features essential for differentiating leukemia subtypes. Figure 9 presents sample prediction images that demonstrate the model's capability to accurately classify leukemia stages.

The experimental results showed that transfer learning models can effectively classify leukemia images with good performance and computational efficiency, as shown in Tables 5–7. Among various models, MobileNetV2 achieved the highest accuracy, 98.36%, with F1-scores of 0.98, indicating strong predictive capability in distinguishing leukemia subtypes. InceptionV3 and VGG16 performed well, with 97.34% and 97.96% accuracy, respectively, while ResNet50 attained 85.89% accuracy. EfficientNetB0 underperformed at 30.88% accuracy, highlighting the importance of model selection. MobileNetV2, with 3.5 million parameters and 29 ms per step, is a lightweight yet highly accurate model for leukemia image classification. The model, when trained with a 5-fold, obtained a better accuracy of 98.83%, claiming the neutrosophic-enhanced MobileNetV2 model to be the best.

**Table 5:** Performance analysis among the transfer learning models.

Model	Accuracy (%)	Precision	Recall	F1	AUC
ResNet50	85.89	0.85	0.84	0.84	0.98
VGG16	97.96	0.97	0.97	0.97	0.99
EfficientNetB0	30.88	0.26	0.31	0.20	0.5
InceptionV3	97.14	0.97	0.96	0.97	0.99
MobileNetV2	98.36	0.98	0.98	0.98	0.99

**Table 6:** Performance analysis among the transfer learning models for 5-fold validation.

Model	Accuracy (%)	Precision	Recall	F1	AUC
ResNet50	84.00	0.84	0.84	0.82	0.97
VGG16	97.76	0.97	0.97	0.97	0.99
EfficientNetB0	28.07	0.11	0.26	0.13	0.53
InceptionV3	97.05	0.96	0.96	0.96	0.99
MobileNetV2	98.83	0.98	0.98	0.98	0.99

**Table 7:** Complexity analysis among the transfer learning models.

Model	Parameters (M)	Time (ms/step)
ResNet50	25.6	60
VGG16	138	90
EfficientNetB3	12	38
InceptionV3	23.9	55
MobileNetV2	3.5	29

All models were trained using the Adam optimizer with a learning rate of 0.001 for 50 epochs and a batch size of 32 as mentioned in Table 8. The convolutional backbone was initialized with ImageNet weights and frozen during training. A global average pooling layer followed by a 128-neuron fully connected layer with ReLU activation and a SoftMax output layer was used for classification.

**Table 8:** Hyperparameter tuning details.

Optimizer	Adam
Learning rate	0.001
Loss function	Categorical cross entropy
Batch size	32
Epochs	50
Class imbalance	Class weight
Weight initialization	ImageNet pre-trained weights
Fine-tuning strategy	Convolutional base: Frozen

## 6 Comparative analysis with state-of-the-art architecture

Experimental results show that the proposed neutrosophic enhancement method significantly outperforms conventional approaches in classification accuracy. This improvement is mainly due to its ability to preserve cellular boundaries and morphological structures, enhance contrast in diagnostically important regions, reduce noise without losing critical features, and effectively manage domain-specific uncertainty in microscopic images. Table 9 compares the proposed approach with existing leukemia detection methods, highlighting the advantages of the proposed approach.

**Table 9:** Comparison with the existing methodology.

References	Architecture	Dataset	Technique	Accuracy (%)
Sulaiman et al. (2023)	MobileNetV2	ALL dataset	MobileNetV2 + RF + SVM	87.8
Abd El-Ghany et al. (2023)	DenseNet	ALL dataset	DL	96.5
Abd El-Ghany et al. (2023)	Logistic regression	ALL dataset	Statistical method	89.0
Vamsi Kumar and Isbella (2023)	EfficientNet-B3	ALL dataset	Transfer learning	97.57
Proposed work	Neutrosophic-enhanced model	ALL dataset	Neutrosophic + MobileNetV2	98.36

## 7 Generalizability analysis

**Figure 10:**
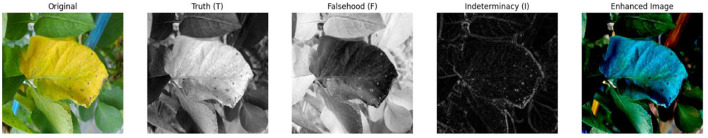
Neutrosophic image decomposition and enhancement of a potato leaf image.

To strengthen the validity, reproducibility, and generalizability of the proposed methodology, additional experiments were conducted using a publicly available potato leaf disease dataset (Shabrina et al., 2023). The dataset comprises six classes: bacteria, fungi, healthy, nematodes, pests, phytophthora, and viruses captured under uncontrolled real-world conditions. The total number of images trained from the dataset is 3,076. The results obtained for Truth, Falsity, Indeterminacy, and Enhanced images are shown in Figure 10.

**Figure 11:**
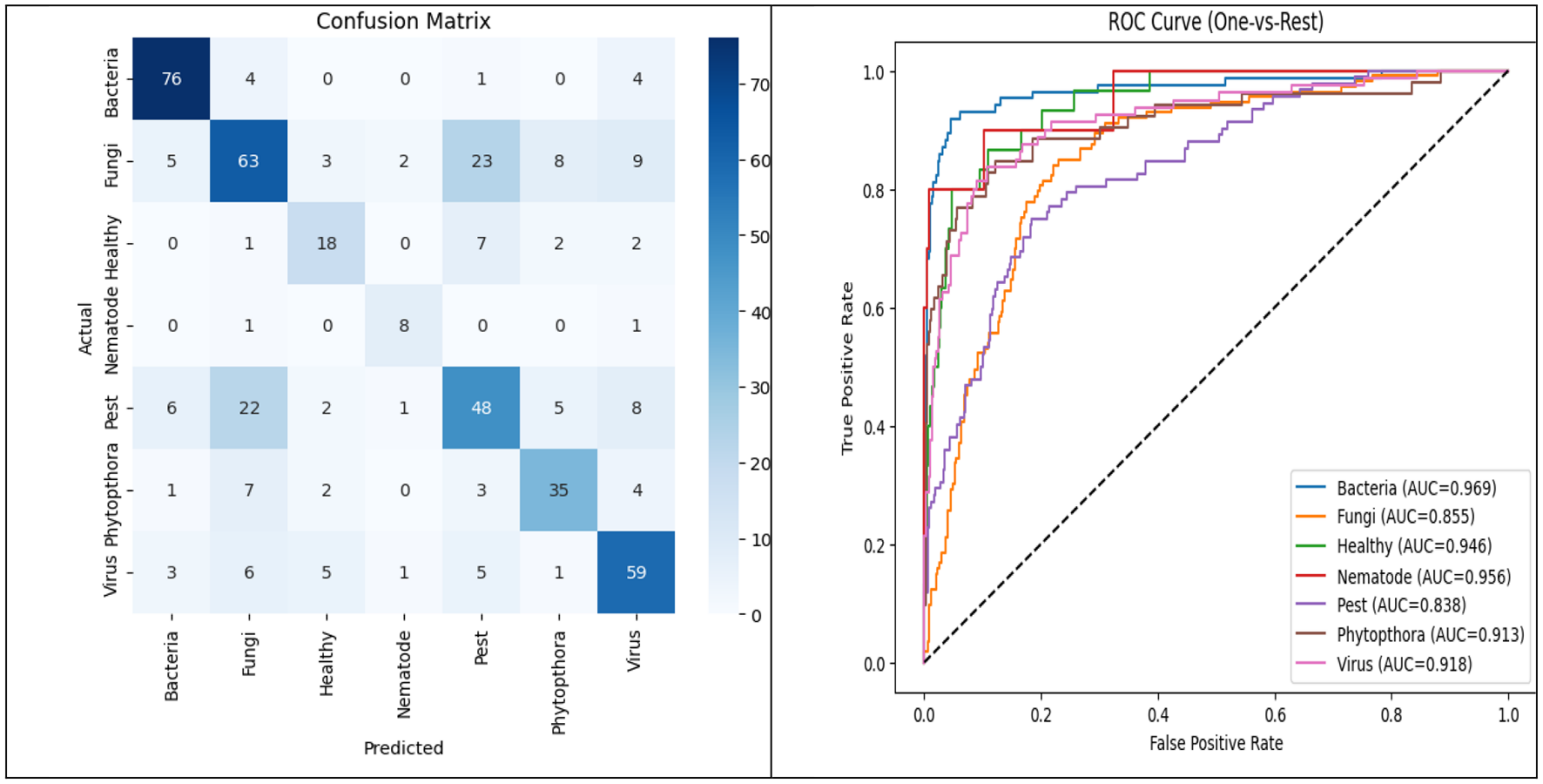
Confusion matrix and ROC potato leaf image.

The model was trained for 50 epochs, and the obtained confusion matrix and ROC plot are given in Figure 11.

The confusion matrix confirms consistent per-class recognition with low false-positive and false-negative rates, while the multi-class ROC analysis exhibits high AUC values, indicating excellent discriminative power and stable generalization performance across all disease categories.

The proposed model achieved a training accuracy of 99.91%, with a sensitivity of 0.99 and an F1-score of 0.99, indicating highly effective feature learning and class discrimination as shown in Table 10.

**Table 10:** Per-class performance metrics.

Class	Precision (%)	Recall (%)	F1-score (%)
Bacteria	1.00	1.00	1.00
Fungi	1.00	1.00	1.00
Healthy	1.00	1.00	1.00
Nematode	0.98	1.00	0.99
Pest	1.00	1.00	1.00
Phytophthora	1.00	1.00	1.00
Virus	1.00	1.00	1.00
Accuracy	99.91		

## 8 Conclusion

The proposed study presented a leukemia cell classification model that integrated deep learning with neutrosophic domain transformation. The decomposition of leukemia images into Truth (T), Indeterminacy (I), and Falsity (F) components enhanced feature representation by maintaining cellular features, thus improving the visualization of diagnostically relevant patterns. The enhanced images were trained using a MobileNetV2 architecture and several transfer-learning models to classify four leukemia categories. This model achieved an overall accuracy of 98.36%, outperforming conventional approaches in precision, recall, and F1-score across all classes. These results demonstrate that neutrosophic preprocessing significantly improved diagnostically relevant cellular features, thereby improving classification performance. Future research can extend the framework to heterogeneous datasets, including multiple microscopy methods and patient groups, such as leukemia subtypes. Attention mechanisms, hybrid transformer CNN architectures, or combined neutrosophic features with clinical parameters and genetic markers can be analyzed for better results. Artificial intelligence models based on neutrosophic components could enable the development of fully automated, end-to-end hematology diagnostic systems and personalized treatment planning. Integrating explainable AI techniques could provide interpretability for clinical decision-making and enable the investigation of multi-modal data fusion.

## Data Availability

The original contributions presented in the study are included in the article/supplementary material, further inquiries can be directed to the corresponding author.

## Footnotes

1 Home Browse Journal Info Submit Manuscript Editorial Board Indexing and Abstracting Guide For Authors Publication Ethics Editor Page Reviewer Page Open Access Policy Unrestricted Reuse in Compliance with BOAI Self-Archiving Policy Publisher Business Model Sponsor and Publisher Contact Us Applications of Neutrosophic Logic in Image Processing: A Survey.
